# RF-KNN-Assisted Local Gaussian Process Regression for Heat Transfer Coefficient Prediction in Hot Strip Coiling Temperature Control

**DOI:** 10.3390/ma19143096

**Published:** 2026-07-18

**Authors:** Dong Chen, Zhenlei Li, Jian Kang, Guo Yuan

**Affiliations:** State Key Laboratory of Digital Steel, Northeastern University, Shenyang 110819, China

**Keywords:** hot strip rolling, heat transfer coefficient, local Gaussian process regression, similar case retrieval

## Abstract

Accurate prediction of the heat transfer coefficient is essential for improving the coiling temperature control in hot strip rolling, especially under frequent rolling condition changes. Conventional layer-based self-learning methods may lead to boundary discontinuities, insufficient sample support for new gauges, and limited information sharing among similar operating conditions. To address these limitations, this paper proposes a random-forest (RF) and K-nearest-neighbor (KNN)-assisted local Gaussian process regression framework for the heat transfer coefficient in hot strip rolling. In the proposed method, RF is first used to select key variables and guide similar-case retrieval. KNN is then employed to retrieve the historical strips most similar to the current strip and to construct a local sample space. Instead of directly using conventional distance-weighted averaging, Gaussian process regression (GPR) is established on the retrieved local samples to model the nonlinear relationship between the process variables and the heat transfer correction coefficient. The proposed method outperforms conventional KNN-based weighting methods in terms of all the evaluation metrics for both first coils and in-lot coils at different speeds. Industrial validation shows that the measured coiling temperature is controlled within ±20 °C over more than 96.5% of the coil length. The results demonstrate that the proposed framework improves the adaptability and online compensation capability of the controlled cooling temperature models.

## 1. Introduction

Hot strip rolling is one of the most important processes in steel production, and controlled cooling in the runout table is crucial to obtain the desired microstructure and properties, which directly affects the strip’s mechanical properties [1]. Temperature control is essential in the strip cooling process because the coiling temperature and cooling path strongly influence phase transformation, grain refinement, and precipitation behavior, thereby determining the strip mechanical properties. Therefore, improving the accuracy and stability of temperature prediction and control is of great significance for ensuring product quality, reducing process variability, and enhancing the overall performance consistency of hot rolling strips [2]. Accordingly, establishing a high-precision temperature prediction model has become an important research topic in both academia and industry.

The temperature mathematical model is used to describe the temperature evolution during controlled cooling of the hot rolling strip. Temperature prediction models are generally constructed based on heat transfer theory and solved by establishing heat-conduction differential equations [3]. At present, one-dimensional finite-difference models are widely used to calculate the strip temperature during hot strip rolling. For the temperature prediction model, obtaining an accurate heat transfer coefficient is a prerequisite for high-precision prediction [4]. Considering the complexity of the boundary conditions and rolling conditions, according to key parameters in the calculation process of the heat transfer coefficient and the actual data generated during the operation, traditional statistical theory methods or other optimization algorithms can be used to optimize the model parameters, so as to realize the accurate calculation of the heat transfer coefficient in the complex heat transfer process and improve the accuracy of temperature control [5]. To decrease the deviation between the calculated strip temperature and the measured value at the coiler entrance, self-learning is used to correct model calculation errors [6]. For strip steel with different materials, thicknesses, etc., the parameters affecting strip cooling are divided into multiple categories according to certain rules; strips in the same categories share the same self-learning parameters, and this method is commonly referred to as layer-based self-learning. However, the number and boundaries of the layers limit the accuracy of the heat transfer coefficient, making accurate prediction difficult, especially when the rolling condition changes significantly.

With the development of computer technology, various advanced algorithms represented by data-driven artificial intelligence technology have gradually become efficient tools to improve control levels in various industries and fields. In order to further improve the control accuracy and product quality, artificial intelligence technology has also received extensive attention and research in the field of the controlled cooling process. Li et al. [7] utilized the T-S fuzzy model to depict the nonlinear components in the controlled cooling process. Colla et al. [8] used BP neural network to predict the parameters of the controlled cooling model by inputting strip parameters and rolling parameters, and realized precise control of the cooling temperature. Xie et al. [9] used linear regression analysis and BP neural network to correct the prediction error of the traditional model. Barrios et al. [10,11] compared 24 intelligent models, such as the neural network model, fuzzy control, and the grey box model to predict the entrance temperature of hot finishing rolling and achieved a prediction accuracy of ±20 °C. Zheng et al. [12] proposed an instance-based learning method of local linear reconstruction. After obtaining similar sample data by distance similarity, the local linear reconstruction method is used to obtain the optimal linear combination and weight coefficient of the current sample and similar samples. After obtaining the weight coefficient, the proportion of each similar sample in the calculation of new samples can be determined. Xing et al. [13] proposed a hybrid genetic algorithm and grey case-based reasoning (GCBR), which uses the grey correlation degree to find similar cases of the current case, and then the hybrid genetic algorithm to find the weight coefficient of similar cases, so as to obtain the best current case output. Zhang et al. [14] proposed a variable scale grid model (VSG) for temperature control. By considering the real influence degree of each factor on the heat transfer coefficient, a variable step size multi-dimensional space for solving the heat transfer coefficient value was established. At the same time, the clustering algorithm was used to finitely grid the space, which greatly improved the calculation efficiency compared with the traditional case-based model and made the model more suitable for industrial applications. In addition, algorithms such as the analytic hierarchy process [15] (AHP) are also widely used in determining attribute weights.

In hot strip rolling, the changing of rolling conditions such as the product grade change, specification change, and roll change will lead to obvious unsteady characteristics. Nevertheless, conventional KNN prediction usually estimates the target value by distance-weighted averaging. The simple linear combination cannot fully describe local nonlinear relationships among process variables and cannot provide prediction uncertainty. Gaussian process regression (GPR), as a Bayesian nonparametric method, can model nonlinear mapping with limited samples and provide both posterior mean and variance [16]. Therefore, this study proposes a random forest and KNN-assisted local Gaussian process regression framework for heat transfer coefficient prediction in the strip cooling process. For each strip, the KNN method is first employed to identify the most relevant strips from the database. Then, a local GPR model is constructed based on the selected similar strips to capture nonlinear relationships within the local operating region. In this way, the proposed method preserves the locality advantage of KNN while enhancing its nonlinear modeling capability and enabling uncertainty quantification.

## 2. Controlled Cooling Process and Mechanism Model

### 2.1. Description of the Controlled Cooling Process

**Figure 1:**
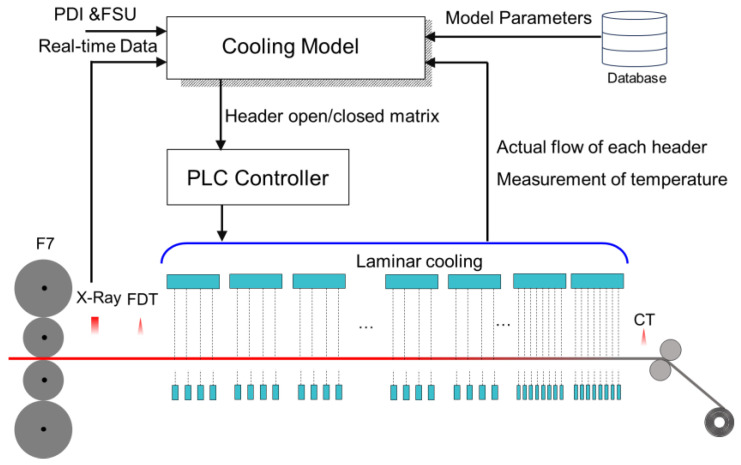
Schematic diagram of a controlled cooling line for hot rolling strips.

Figure 1 shows a controlled cooling line for hot strip rolling. After finishing rolling, the strip enters the runout table and is cooled by several cooling banks. In practice, the system controls the temperature through three steps. In step 1, the system calculates the initial cooling header open/closed matrix based on the primary input data and finishing mill setup data. After the strip completes threading, step 2 is triggered when the actual finishing mill delivery temperature (FDT) is detected. In step 2, the system controls the headers, open or closed, with the real-time data of the FDT and strip velocity. The sensors measure the strip FDT and velocity every 200 ms and send the data to the control system, so step 2 could be triggered every 200 ms. Considering the space between the cooling banks and costs, only the start temperature (FDT) and final coiling temperature (CT) are available for temperature control and a feedback controller is needed. As shown in Figure 1, the feedback controller will be running in step 3 when the strip head passes through the CT pyrometer (Optris GmbH & Co. KG, Berlin, Germany).

### 2.2. Temperature Mathematical Model

To accurately describe the temperature evolution of the strip during controlled cooling, a temperature prediction model is established by analyzing the heat transfer characteristics in the strip cooling process and heat balance of a moving strip element. Because the strip length is much larger than its thickness and width, and the strip velocity in the longitudinal direction is relatively high, heat conduction along the rolling direction can be neglected. Therefore, the heat transfer problem during strip cooling can be simplified as a one-dimensional transient heat-conduction problem along the thickness direction, as expressed in Equation (1):

(1) $$\frac{\partial }{\partial z}\left(\lambda \frac{\partial T}{\partial z}\right)=\rho c_{p}\frac{\partial T}{\partial \tau }$$

where T is the strip surface temperature, K; τ represents time, s; λ means strip thermal conductivity, W/(m·K); ρ is steel density, kg/m^3^; cp represents specific heat capacity, J/(kg·K); z means the strip thickness direction.

The boundary conditions on the top and bottom surfaces can be described in a form like Newton’s convection Equation (2), and equivalent to water cooling and air cooling:

(2) $$\pm \lambda \frac{\partial T}{\partial z}=h\left(T-T_{\infty }\right)$$

where h is the heat transfer coefficient, W/(m^2^·K); the cooling medium is either water or air, so the $T_{\infty }$ in the boundary conditions should be the cooling water temperature or air temperature.

In industrial applications, to simplify calculations, theoretical statistical models are typically employed to derive the equivalent heat transfer coefficient of water-cooling convection heat transfer. The strip enters the cooling zone at a certain speed, and its water-cooling process is affected by multiple factors such as strip speed, strip temperature, cooling water flow rate, cooling water temperature and water pressure. Hence, the theoretical statistical model for the equivalent convective heat transfer coefficient of water cooling can be established, presenting as follows [4]:

(3) $$h_{w}=A_{1}\cdot \frac{F}{BL}\cdot t^{A_{2}}\cdot \exp\left(-A_{3}\left(T-T_{w}\right)\right)\cdot {\left(\frac{V}{V_{B}}\right)}^{A_{4}}{\left(\frac{W_{aB}}{W_{a}}\right)}^{A_{5}}{\left(\frac{P}{P_{B}}\right)}^{A_{6}}$$

where A1–A6 are model coefficients, F is total water flow in m^3^/h, T_w_ is water temperature in K, P is water pressure in MPa, V is the actual velocity of the strip in m/s, P_B_ is reference water pressure in MPa, V_B_ is reference velocity in m/s, W_B_ is reference water temperature in K, B is width of strip in m, L is effective cooling length in m, and t is strip thickness in m.

### 2.3. Heat Transfer Correction Coefficient with Self-Learning

The controlled cooling process of the hot rolled strip has typical strong nonlinearity and strong coupling characteristics, and its boundary conditions change dynamically with strip temperature, running speed, cooling water state, surface scale, phase transformation heat release, and radiation heat transfer, so it is difficult to completely and accurately describe the actual heat transfer behavior by a single mechanism model. Therefore, in industrial control systems, it is usually necessary to introduce heat transfer correction coefficients or adaptive coefficients on the basis of mechanism models to compensate for prediction deviations caused by model assumptions, boundary simplification, and field disturbances. For this purpose, an adaptive coefficient is introduced into the water-cooled convective heat transfer model:

(4) $$h_{w}=\alpha \cdot A_{1}\cdot \frac{F}{BL}\cdot t^{A_{2}}\cdot \exp\left(-A_{3}\left(T-T_{w}\right)\right)\cdot {\left(\frac{V}{V_{B}}\right)}^{A_{4}}{\left(\frac{W_{aB}}{W_{a}}\right)}^{A_{5}}{\left(\frac{P}{P_{B}}\right)}^{A_{6}}$$

where *α* is the correction coefficient of the heat transfer coefficient. However, in real conditions, due to the disturbance of multiple influencing factors, how to obtain the accurate adaptive coefficient is the key to achieving high-precision temperature control.

In practice, the heat transfer correction coefficient is usually obtained by a self-learning strategy. The basic principle is to calculate the actual temperature drop by using the measured temperature of FDT and CT and calculate the theoretical temperature drop with a temperature model. Then, according to the deviation between the actual temperature drop and the temperature drop calculated by the model, the water-cooling heat transfer coefficient is corrected. The method has a simple structure, convenient engineering implementation, and can effectively compensate model error and field disturbance under stable production conditions. However, the applied self-learning strategy still has obvious limitations. First, self-learning coefficients are usually stored and called hierarchically according to rules such as steel grade, thickness, width, target temperature, or cooling mode, which is essentially a discrete parameter management method. Second, with the increase of product specifications and steel grades, the number of layers expands rapidly, and some new steel grades with new specifications or first coils of steel lack sufficient historical samples, resulting in insufficient reliability of the self-learning coefficients. Third, the traditional method mainly relies on the historical average or exponential smoothing within the same layer, so it is difficult to make full use of the historical experience of similar rolling conditions among different layers, and it is also difficult to describe the continuous influence of strip velocity change on the heat transfer correction coefficient. Therefore, the adaptability and the prediction accuracy of the traditional layer table, self-learning method are limited under complex rolling conditions such as variable speed rolling and new specification switching.

## 3. Proposed Method

### 3.1. Overall Framework

To solve the above problems, this paper uses the heat transfer correction coefficient obtained by self-learning as the supervised learning label and constructs an RF-KNN-assisted local Gaussian process regression for the heat transfer coefficient. First, random forest is used to evaluate the importance of each parameter for the heat transfer coefficient. Key input variables are then selected to prevent weakly correlated and redundant variables from interfering with similar-case retrieval. Then, according to the specifications, temperature, speed, cooling conditions, and composition information of the current strip, the samples most similar to the current rolling conditions are retrieved from the historical database to construct a local training set. Finally, Gaussian process regression is used to model the selected local samples and predict the heat transfer coefficient under the current rolling conditions. In this way, the method in this paper no longer relies on the fixed layer table but dynamically extracts effective information from historical samples based on continuous similarity, thus improving the adaptability of the model.

### 3.2. Similar Case Retrieval Based on RF-KNN

Random forest is used to estimate the relative importance of candidate variables [17]. Variables with high cumulative importance scores are retained for subsequent KNN-based retrieval, while weakly relevant variables are removed to reduce noise and computational burden. Feature selection is performed only on the training set to avoid information leakage. Based on the input information of the current strip, the historical strips most similar to the current strip are retrieved from the database. Then, the heat transfer correction coefficient α corresponding to the current strip is calculated according to the information of the similar strips. According to the parameters of the mechanistic model and expert experience, factors such as strip composition, strip specification, strip speed, cooling water temperature, cooling water pressure, and cooling mode all have certain effects on the overall surface heat transfer coefficient during strip cooling. The specific parameters involved are listed in Table 1.

In the process of similar strip retrieval, according to the input parameters of the current strip Xn + 1 to be predicted, the similarity between the current strip Xn + 1 and the historical strip Xi in the database is calculated, and the k strip data most similar to the current strip are found. Since there is no actual temperature data of the current strip, the target finishing rolling temperature and the target coiling temperature are used as the actual temperatures for the similar strip retrieval. On the other hand, as shown in Table 1, the dimensions of different parameters of strip steel are quite different, so it is necessary to normalize the data. To improve the adaptability of the model to new specifications and new steel grades, the maximum and minimum values of each influencing parameter are limited according to manual experience. The specific normalization calculation method is shown in Equation (5):

(5) $$x^{*}=\frac{x-x_{\min}}{x_{\max}-x_{\min}}$$

where x, x_min_, x_max,_ and x^∗^ represent the sample data, the minimum value of the sample data set, the maximum value of the sample data set, and the normalized sample data respectively.

Because the influence degree of different input parameters on the cooling process of the strip is different, the local similarity weight vector H is added in the global similarity calculation in this part to describe the influence degree of different input parameters on the cooling process. The distance Dis (Xn + 1, Xi) between the strip Xn + 1 and the sample Xi can be expressed as follows:

(6) $$Dis\left(X_{n+1},X_{i}\right)=\sum_{j=1}^{l}H{\left\|x_{i,j}-x_{n+1,j}\right\|}_{2}$$

When the similar strip of the current strip is found, the information of the similar strip can be used to calculate the heat transfer correction coefficient of the current strip. The smaller the distance from the current strip, the greater the similarity, and it should occupy a greater weight in the prediction of the heat transfer correction coefficient; on the contrary, the farther the distance, the smaller the weight should be, accordingly. After obtaining the weights of k similar strips, the heat transfer correction coefficient of the current strip can be calculated according to the following Equation (7):

(7) $$y_{N+1}=\sum_{m=1}^{k}W_{m}y_{m}$$

where *y*_N+1_ is the heat transfer correction coefficient of the current strip, *y*_m_ represents the heat transfer correction coefficient of the similar strip m, and Wm represents the weight of the similar strip m.

**Figure 2:**
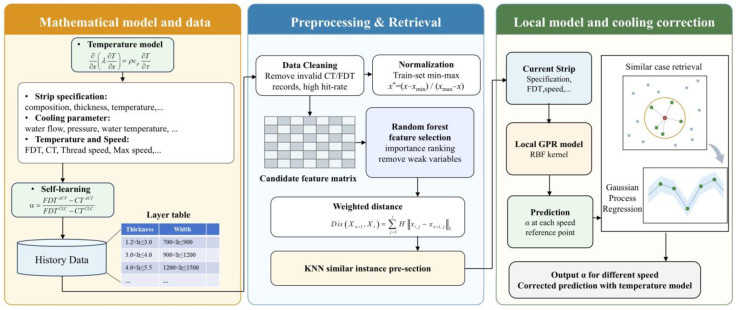
Flowchart of RF-KNN-assisted local Gaussian process regression framework.

As shown in Figure 2, to accurately predict the heat transfer correction coefficient at different speeds, the strip speed running interval is divided into multiple speed intervals according to certain rules, and multiple speed reference points are set. Based on the heat transfer correction coefficient of the speed reference point, the heat transfer correction coefficient at different speeds can be obtained through interpolation and extrapolation, so as to accurately predict the heat transfer coefficient in the rolling process and realize the temperature prediction and control of the whole length of the strip. Generally speaking, the rolling speed of the thick gauge strip is slow, but it is sensitive to speed change, so it is divided into denser sections in the low-speed sections; However, for the high-speed section where thin-gauge production is located, a sparse section is set.

### 3.3. Local Gaussian Process Regression Algorithm

GPR is a Bayesian nonparametric regression method that is well suited to nonlinear modeling under limited and uncertain industrial data. Unlike conventional parametric models, GPR does not assume a fixed analytical form of the mapping function. Instead, the unknown function is regarded as a random function, and any finite set of its function values is assumed to follow a joint Gaussian distribution. Therefore, GPR can describe the nonlinear relationship between the process variables and the target output through a prior distribution defined by a mean function and a covariance function.

After obtaining the k similar cases, the heat transfer correction coefficient of the current strip can be expressed as a linear combination of the correction coefficients of the similar strips. However, it is essentially a distance-based local weighted averaging method, which assumes that the influence of each similar sample on the current sample is determined only by its distance, making it difficult to further characterize the complex nonlinear relationships among local samples. It cannot provide uncertainty information for the prediction results. Therefore, when local disturbances, noise, or multivariable coupling effects still exist among the similar samples, using only the weighted linear combination may limit the prediction accuracy of the heat transfer correction coefficient.

To address this issue in this paper, the k similar cases retrieved by KNN are not directly used for simple weighted averaging as the final prediction result. Instead, these k similar samples are used as a local training set to further construct a local Gaussian process regression model. KNN is responsible for selecting the local sample space that is most relevant to the current rolling condition from the historical database, while GPR is used to establish a nonlinear probabilistic regression model within this local sample space, thereby obtaining the predicted heat transfer correction coefficient of the current strip. In this way, the proposed method preserves the locality of similar-case reasoning while improving the ability to represent nonlinear relationships and prediction uncertainty.

In this paper, the radial basis function (RBF) kernel is selected as the covariance function of the Gaussian process, and its expression is as follows:

(8) $$K\left(X,X^{\prime }\right)=\sigma_{f}^{2}\exp\left(-\left\|\frac{X-X^{\prime }}{2l^{2}}\right\|\right)$$

The mean function of the local GPR model is set as a constant mean function, which represents the local average level of the heat transfer correction coefficient within the selected similar samples. The radial basis function (RBF) kernel is adopted as the covariance function because the heat transfer correction coefficient usually varies smoothly with strip specification, temperature, velocity, and cooling conditions in a local rolling-condition space. Other kernel functions, such as the linear kernel, can be further compared in future work, but the RBF kernel is used as the baseline kernel in this paper to balance model flexibility and computational efficiency.

### 3.4. Prediction Model Evaluation Metrics

To evaluate the accuracy of the prediction model, root mean square error (RMSE), mean absolute error (MAE), mean absolute percentage error (MAPE), and coefficient of determination (R^2^) were selected as the evaluation metrics. Among these metrics, RMSE, also known as the standard error, is highly sensitive to extremely large or small errors during the prediction process and thus can well reflect the prediction precision. MAE refers to the average of the absolute values of deviations between all predicted values and actual values; it can effectively avoid the offsetting of errors and therefore accurately reflect the magnitude of the actual prediction errors, especially showing high sensitivity to outliers. MAPE takes into account the ratio of the error between the predicted value and the true value to the true value, with a smaller value indicating a better regression performance of the model. The evaluation indicators were calculated as follows:

(9) $$\mathrm{RMSE}=\sqrt{\frac{\sum_{i=1}^{N}{\left({\hat{y}}_{i}-y_{i}\right)}^{2}}{N}}$$

(10) $$\mathrm{MAE}=\frac{1}{N}\sum_{i=1}^{N}\left|{\hat{y}}_{i}-y_{i}\right|$$

(11) $$\mathrm{MAPE}=\frac{1}{N}\sum_{i=1}^{N}\frac{\left|{\hat{y}}_{i}-y_{i}\right|}{y_{i}}\times 100\%$$

(12) $$R^{2}=1-\frac{\sum_{i=1}^{N}{\left(y_{i}-{\hat{y}}_{i}\right)}^{2}}{\sum_{i=1}^{N}{\left(y_{i}-\bar{y}\right)}^{2}}$$

To rigorously evaluate model performance from an industrial application perspective, coefficient accuracy was adopted as an additional assessment metric. This metric explicitly accounts for industrial requirements, in which prediction errors within a specified tolerance are acceptable for operational decision-making. A prediction is considered correct if the absolute deviation between the predicted and reference coefficient is within the tolerance range Δ:

(13) $$\left|{\hat{y}}_{i}-y_{i}\right|\leq \Delta$$

where Δ means the industry-defined tolerance range, usually 0.05. For an actual coefficient of 0.85, predicted values within the interval [0.80, 0.90] are considered acceptable.

The batch prediction accuracy is then calculated across a production batch of size N as follows:

(14) $$\mathrm{Accuracy}=\frac{\sum_{i=1}^{N}\prod \left(\left|{\hat{y}}_{i}-y_{i}\right|\leq \Delta \right)}{N}$$

where $\prod \left(\left|{\hat{y}}_{i}-y_{i}\right|\leq \Delta \right)$ is the indicator function that returns 1 if the condition is satisfied, and 0 otherwise.

## 4. Results and Discussion

The proposed method is evaluated using production data from an industrial hot strip rolling line. The inverse-distance weighting method (IDW) and Gaussian-function-assigned weighting method (GFAW) were used as comparison benchmarks.

### 4.1. Feature Selection Results

During the controlled cooling process, the comprehensive heat transfer coefficient is affected by various process parameters. If all the historical parameters are directly input into the prediction model, it will not only increase the complexity of the model but also introduce redundant variables and weakly correlated variables, thus reducing the generalization ability and prediction accuracy of the model. Therefore, in this paper, the combination of random forest was used to screen the input features to determine the correction coefficient of heat transfer.

**Figure 3:**
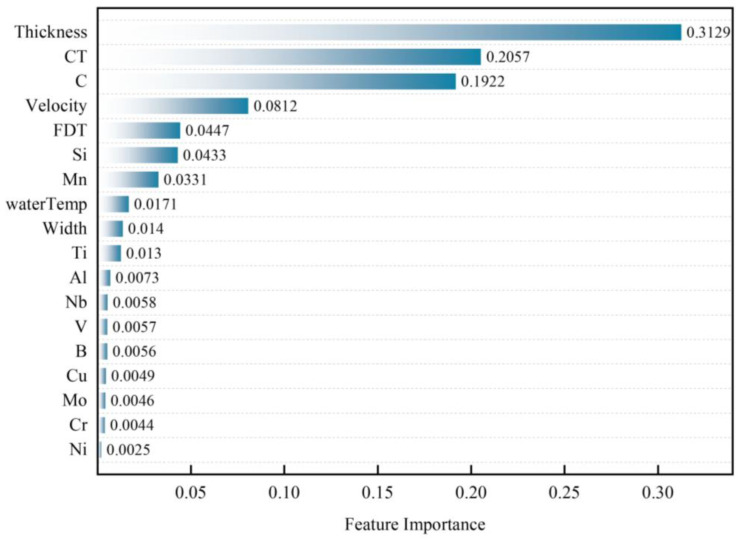
Importance ranking of influencing parameter for the controlled cooling process.

After obtaining the optimal parameters, the random forest algorithm was employed to rank the importance of factors affecting the cooling process in the dataset, with the ranking results presented in Figure 3. Before random forest screening, cooling mode and cooling water pressure were directly retained as input variables because of their explicit and significant effects on the controlled cooling process. Therefore, they were not included in the feature screening procedure. The results in Figure 3 indicate that strip thickness has the highest importance score, indicating that it has the most significant effect on the controlled cooling process. This is followed by the strip final cooling temperature, namely the coiling temperature. The content of element V shows the relatively smallest influence on the strip cooling process. It can also be clearly observed from the feature importance ranking results that the cumulative importance score of nine parameters (including strip thickness, strip speed, and coiling temperature) accounts for 95% of the total score. In contrast, the importance scores of the microalloying elements in the steel (such as Al, Nb, V, B, Cu, Mo, Cr, and Ni) are all below 0.01, indicating their minor effects on the strip cooling process. Therefore, to reduce model complexity and computational load when constructing a prediction model for the key parameters of the controlled cooling process, the aforementioned chemical element features can be excluded from the similar case indexing.

Given the favorable application of the back propagation neural network (BPNN) algorithm in predicting the key parameters of hot-rolled steel, in order to verify the effectiveness of the key variable screening results for the controlled cooling process based on random forest and combined with the practice of mechanism models, this paper adopts a stepwise increment method to select key variables with higher feature importance rankings as input parameters and establishes BPNN based water flow prediction models for the strip cooling process, respectively. The MSE and R^2^ were used as indicators to evaluate the accuracy of the models, and the feature importance of the key variables is ultimately determined according to the water flow prediction accuracy.

**Figure 4:**
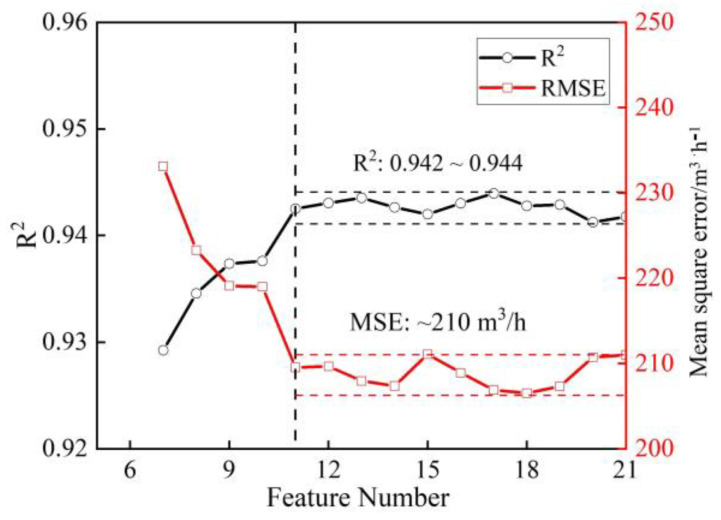
Result of flow prediction with different feature numbers.

First, the cooling mode and cooling water pressure were selected as the input parameters of the model. Then, based on the feature importance ranking results, a stepwise increment method was adopted in descending order of feature importance, with a minimum of 5 and a maximum of 17 key parameters selected to establish water flow prediction models for the cooling process, respectively. The prediction results are shown in Figure 4. The research findings indicate that with the increase in the number of selected key variables, R^2^ of the water flow prediction model increases gradually while the MSE decreases progressively, demonstrating that the prediction accuracy of the model is continuously improving as the number of input parameters grows. When the number of key variables used for modeling is 11 or more, the coefficient of determination and mean squared error of the model tend to stabilize, with the R^2^ value ranging from 0.942 to 0.945 and the MSE value stabilizing at approximately 210 m^3^/h. This shows that on the basis of determining the cooling mode and cooling water pressure as input parameters, selecting the top nine key variables with the highest feature importance to establish the water flow prediction model for the cooling process can achieve stable prediction with high accuracy. This result is consistent with the screening results of the key parameters for the controlled cooling process based on the random forest algorithm.

Therefore, when constructing the heat transfer coefficient prediction model for the controlled cooling process, this paper selects the nine selected variables with the highest feature importance and two retained variables as the baseline process parameters: strip thickness, coiling temperature, carbon mass fraction, strip speed, finishing rolling temperature, silicon mass fraction, manganese mass fraction, titanium mass fraction, and cooling water temperature, with cooling mode and cooling water pressure.

### 4.2. The Heat Transfer Coefficient Prediction with Local Gaussian Process Regression

Considering the actual rolling production process, there may be some differences between the rolling state (heating state, rolling condition) of the first coil steel after changing the steel grade and specification and the similar strip steel recently rolled. This indicates that the coefficient of the first coil steel has no obvious correlation with the rolling time, so the influence of time is not considered in the prediction of the heat transfer correction coefficient of the first coil steel. For the steel coils in the batch, the first steel coil that has been cooled has a strong reference value and can be regarded as a similar strip steel with a larger weight. Therefore, according to the actual production characteristics, in the research process of this paper, the strip data are divided into the first coil steel and the in-lot coils in the batch for research, respectively.

**Figure 5:**
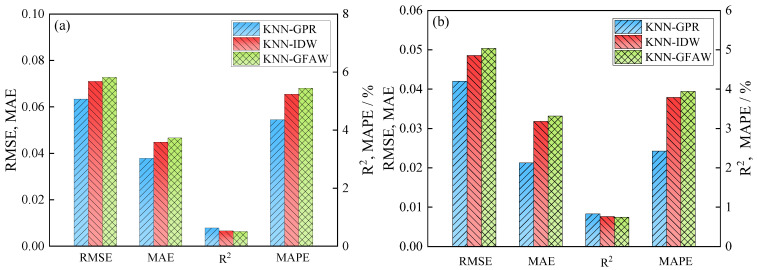
Error histogram of output prediction methods. (**a**) First coil; (**b**) in-lot coil.

Based on industrial field data and production characteristics, the RMSE, MAE, and MAPE of three heat transfer correction coefficient prediction models of GPR-KNN, IDW-KNN, and GFAW-KNN were simulated and compared. As shown in Figure 5, the GPR-KNN-based model achieves significantly better prediction performance for the heat transfer correction coefficient than IDW-KNN and GFAW-KNN for both first coils and in-lot coils. Among them, the prediction errors RMSE, MAE, and MAPE of the heat transfer correction coefficient of the first coil steel by the GPR-KNN model are 0.063, 0.037, and 4.3%, respectively, which are significantly lower than those of IDW-KNN 0.071, 0.045, and 5.2% and GFAW-KNN 0.072, 0.046, and 5.4%. The prediction errors RMSE, MAE, and MAPE of the GPR-KNN model for the in-lot coil heat transfer correction coefficient were 0.042, 0.021, and 2.4%, respectively, which were also lower than those of IDW-KNN 0.048, 0.031, 3.7% and GFAW-KNN 0.050, 0.033, and 3.9%.

**Figure 6:**
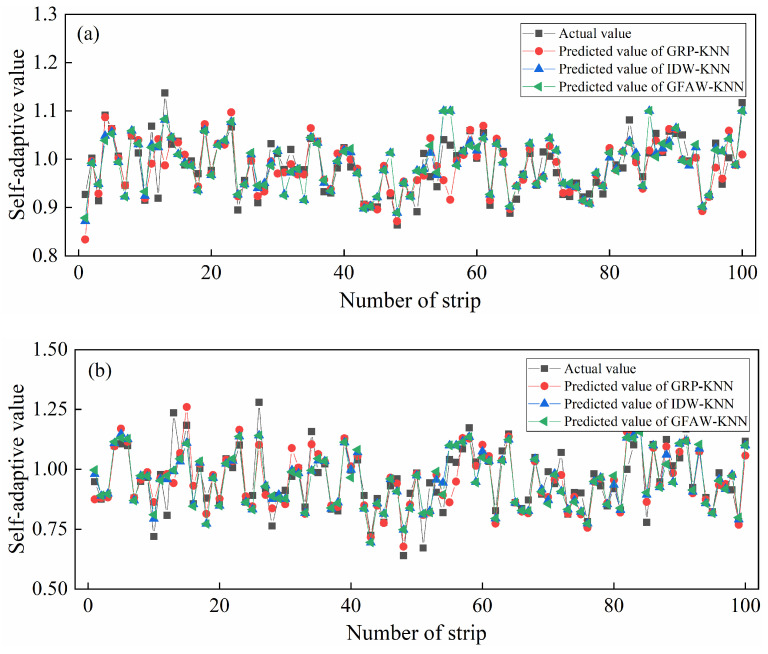
Comparison of predicted values and measured values for first strip based on different models. (**a**) Thread stage; (**b**) running stage.

**Figure 7:**
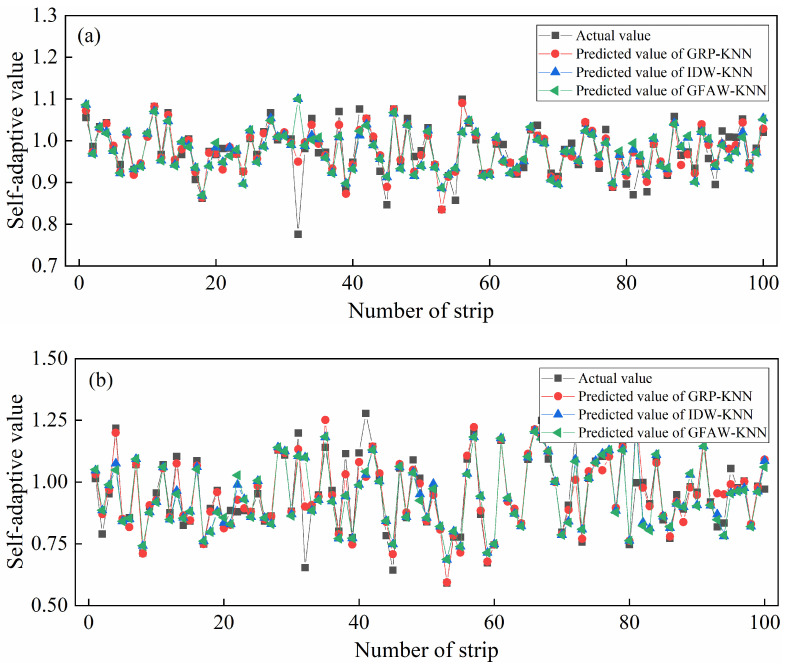
Comparison of predicted values and measured values for strip in-lot based on different models. (**a**) Thread stage; (**b**) running stage.

In order to facilitate visual analysis after the heat transfer correction coefficient of the whole length of strip is predicted by the interpolation method, the prediction results of the heat transfer correction coefficient of the low-speed strip penetration section and the high-speed stable running section of strip are selected, and the prediction accuracy of the heat transfer correction coefficient of the whole length of the strip is analyzed. The predicted and actual values of heat transfer correction coefficients of the three real-time learning models of GPR-KNN, IDW-KNN, and GFAW-KNN are shown in Figure 6 and Figure 7 respectively. As shown in Figure 6 and Figure 7, the prediction model of the heat transfer correction coefficient of the hot rolled strip based on GPR-KNN has a higher prediction accuracy than the other two algorithms in predicting both the first coil and the in-lot strip.

**Figure 8:**
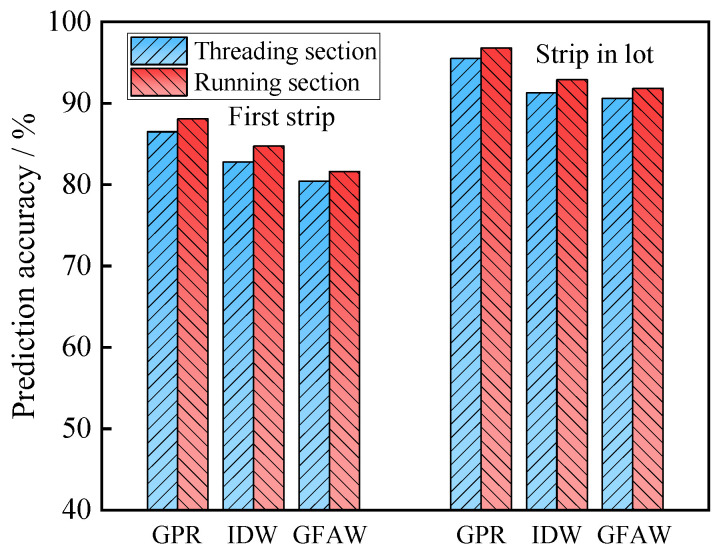
Prediction accuracy of heat transfer coefficient under different models.

The prediction accuracy of the three models, GPR-KNN, IDW-KNN, and GFAW-KNN, is shown in Figure 8. Comparing the prediction results of the heat transfer correction coefficient of the belt-through segment, the prediction accuracy of the GPR-KNN model for the first steel coil and the coil in-lot is 86.5% and 95.5% respectively, which is higher than that of IDW-KNN and GFAW-KNN (82.8% and 91.3%, and 80.4% and 90.6%), which once again proves that the GPR-KNN algorithm has higher prediction accuracy. According to the prediction results of the heat transfer correction coefficient of the high-speed running section, the prediction accuracy of the heat transfer correction coefficient of the high-speed strip section is higher than that of the head strip section, which shows that the proposed heat transfer correction coefficient prediction method of the multi-speed reference point can accurately predict the heat transfer correction coefficient at different speeds, thus providing a good foundation for full-length temperature prediction and control. On the other hand, the prediction accuracy of the heat transfer correction coefficient of the strip head is lower than that of the high-speed section, because in the production process, the adaptive correction value of the whole length of the strip changes with the change of rolling conditions, but when the strip is in the high-speed running section, the feedback function of the control system is started, and the temperature control deviation of the control system becomes smaller. The small temperature deviation makes the variation range of the adaptive value of the high-speed running section smaller than that of the head belt-penetrating section, so under the same allowable absolute deviation, the prediction accuracy of the adaptive value of the high-speed section is higher than that of the low-speed head belt-penetrating section.

**Figure 9:**
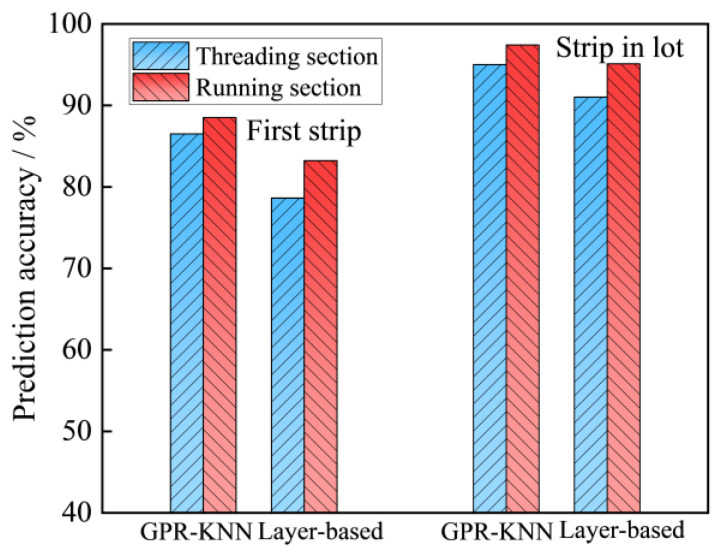
Prediction accuracy histogram comparison between GPR-KNN model and traditional method.

Using the same modeling data, the GPR-KNN method was compared with the traditional stratification layer-based self-learning method. The model parameter prediction accuracy of the two methods is shown in Figure 9. The prediction accuracy of the traditional method in the first steel coil and in-lot strip is 78.6% and 83.2% for the thread selection, 91.9% and 95.1% for the running selection, respectively, which are lower than the proposed GPR-KNN model. Based on this, it can be explained that the prediction effect of the developed GPP-KNN is better than that of the traditional method with the same data background.

### 4.3. Computational Complexity Analysis

To demonstrate the online applicability of the local GPR method, the computational complexity of local GPR is also compared with that of global GPR. For global GPR, all N historical samples are used to construct the covariance matrix. The training complexity is approximately O(N^3^), and the storage complexity is O(N^2^), resulting in high memory consumption and computational cost. Therefore, global GPR is difficult to directly apply to online real-time prediction under large-scale industrial datasets.

**Figure 10:**
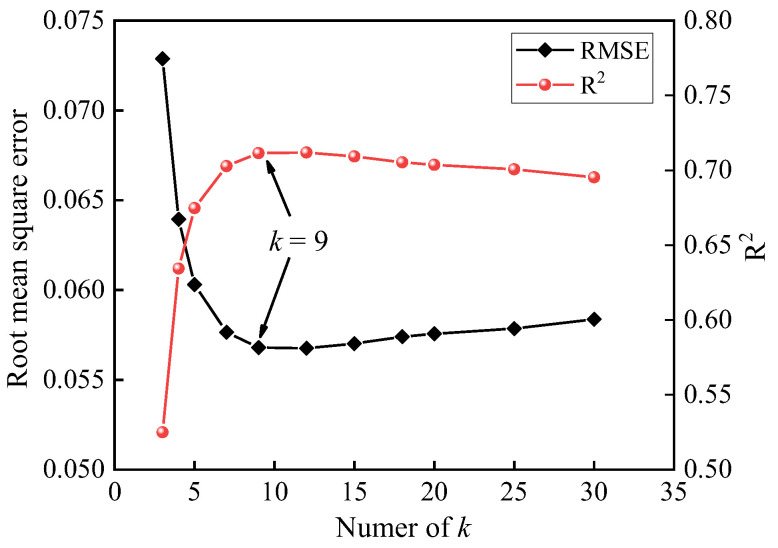
The estimation result of GPR-KNN under different values of *k*.

To determine the appropriate number of similar strip coils k, the relationship between the number of similar strip coils k and the prediction accuracy of the model was studied, and the results are shown in Figure 10. This shows that the number of similar strip coils k has a great influence on the prediction accuracy of the model. With the increase of the k value, the RMSE predicted by the model shows a trend of first decreasing and then increasing, and the determination coefficient R^2^ shows the opposite result. The above results show that too large a k value leads to the screening of more unrelated cases and reduces the prediction accuracy of the model. When the number of similar strip coils is nine, the model has the best prediction accuracy, and the root mean square error RMSE and determination coefficient R^2^ are 0.0568 and 0.718, respectively. Therefore, in this paper, at most nine similar strip coils are selected when constructing the prediction model of the heat transfer correction coefficient. For local GPR, selecting only nine nearest historical samples for each current strip constructs a local GPR model based on these samples. Thus, the training complexity is much less than the global GRP. Therefore, local GPR can significantly reduce the computational burden and prediction time while maintaining the nonlinear modeling capability of GPR, making it more suitable for online real-time prediction in industrial controlled cooling processes.

## 5. Industrial Validation

The proposed local GPR model was implemented and validated on a hot strip mill. The model was integrated into the existing cooling control system to predict the heat transfer coefficient online and compensate for prediction errors in the temperature calculation model during production. In the industrial application, the historical self-learning coefficients were used as target labels, while random forest feature selection, KNN-based similar-strip retrieval, and local Gaussian process regression were performed to generate adaptive correction coefficients for the current strip.

The validation was carried out on typical steel grades, including plain carbon steel, cold rolled substrate, and other representative steel grades. These products cover a wide range of thicknesses, rolling speeds, cooling strategies, and target coiling temperatures, thereby providing a representative basis for evaluating the adaptability of the proposed method. Compared with the conventional layer-based self-learning method, the proposed framework no longer relies on predefined discrete layers. Instead, it dynamically retrieves similar historical cases according to the current rolling condition and establishes a local regression model for heat transfer correction. This improves model adaptability, especially for new strip gauges, first coils after specification changes, and variable-speed rolling conditions.

**Figure 11:**
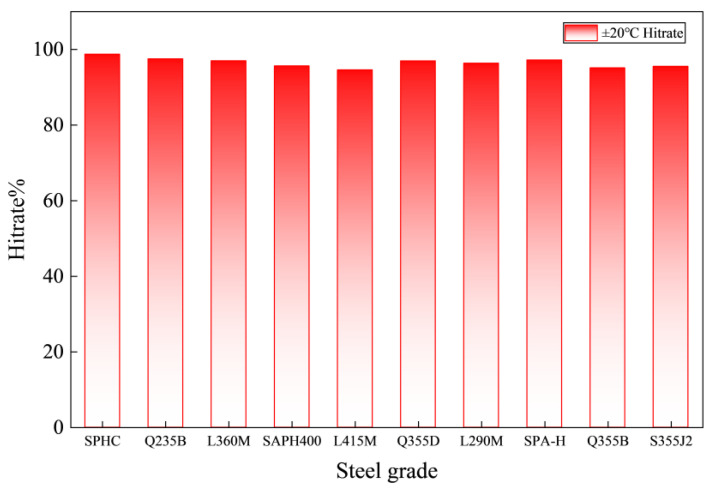
The application of the proposed method in hot strip rolling line.

After applying the proposed correction strategy, the temperature prediction accuracy of the runout table cooling model was markedly improved. The predicted heat transfer correction coefficients showed better agreement with the self-learning reference values, and fluctuations in the temperature error along the strip length were reduced. In industrial production, the measured coiling temperature was controlled within ± 20 °C over more than 96.5% of the coil length as shown in Figure 11. These results demonstrate that the proposed local GPR framework can effectively improve the online compensation capability of the cooling model and enhance the full-length temperature control accuracy in hot strip rolling.

## 6. Conclusions

This paper proposed a RF-KNN-assisted local Gaussian process regression framework for heat transfer coefficient correction in hot strip rolling. The main conclusions are as follows:

(1) Random forest effectively identifies the dominant variables affecting the heat transfer correction coefficient. A total of 11 key parameters with the greatest influence on the controlled cooling process were selected, reducing the interference of weakly related variables in the KNN-based similar-case retrieval.
(2) Compared with IDW-KNN and GFAW-KNN, the proposed method achieves lower RMSE, MAE, and MAPE values for both first coils and in-batch coils. For first coils, the RMSE, MAE, and MAPE are 0.063, 0.037, and 4.3%, respectively. For in-batch coils, the corresponding values are reduced to 0.042, 0.021, and 2.4%, demonstrating superior prediction accuracy under different production conditions.
(3) The analysis of the number of neighboring samples shows that the prediction performance is sensitive to the value of k. When k = 9, the local GPR model achieves the best overall performance. Compared with global GPR, the proposed local modeling strategy significantly reduces the computational complexity and is therefore more suitable for online industrial applications.
(4) Industrial validation on a hot strip rolling line demonstrated that the proposed method improves the online compensation capability of the mechanism-based cooling model. The measured coiling temperature can be controlled within ± 20 °C over more than 96.5% of the coil length.

## Figures and Tables

**Table 1:** Parameters included in strip sample data.

	No.	Parameter	Description	Unit	No.	Parameter	Description	Unit
Input	1	*Dt*	Rolling time	min	11	*Mo*	Content of Mo	%
	2	*t*	Strip thickness	mm	12	*B*	Content of B	%
	3	*W*	Strip width	mm	13	*T_w_*	Water Temperature	K
	4	*C*	Content of C	%	14	*P*	Pressure	bar
	5	*Si*	Content of Si	%	15	*T_F_*	FDT	K
	6	*Mn*	Content of Mn	%	16	*T_C_*	CT	K
	7	*Ni*	Content of Ni	%	17	*V_thd_*	Thread Speed	m/s
	8	*Cr*	Content of Cr	%	18	*V_max_*	Max speed	m/s
	9	*V*	Content of V	%	19	*F*	Water flow	m^3^/h
	10	*Nb*	Content of Nb	%	20	*M*	Cooling mode	—
Output	1	*α*	Heat transfer correction coefficient	—				

## Data Availability

The original contributions presented in this study are included in the article. Further inquiries can be directed to the corresponding authors.

## Nomenclature

RF	Random forest
KNN	K-nearest-neighbor
GPR	Gaussian process regression
APH	Analytic hierarchy process
FDT	Finishing mill delivery temperature
CT	Coiling temperature
RBF	Radial basis function
RMSE	Root mean square error
MAE	Mean absolute error
MAPE	Mean absolute percentage error
R2	Coefficient of determination
IDW	Inverse-distance weighting method
GFAW	Gaussian function assigned weighting method
BPNN	Back propagation neural network
